# Computer-aided analysis for optimal screw insertion in lateral mass of C1: An anatomical study

**DOI:** 10.1007/s00402-017-2678-y

**Published:** 2017-03-29

**Authors:** Renate Krassnig, Jakob Andrea Orlandi, Ellen Tackner, Gloria Hohenberger, Paul Puchwein

**Affiliations:** Department of Orthopedics and Traumatology, Medical University Graz (MUG), Auenbruggerplatz 5, 8036 Graz, Austria

**Keywords:** C1, Cervical spine, Jefferson fracture, Lateral mass screw

## Abstract

**Introduction:**

Motion preserving techniques in C1 ring fractures are increasingly used especially in young patients. Therefore, lateral mass screws are inserted in the first vertebra and connected by a rod. The purpose of this study was to determine safe zones regarding the vertebral arteries and the medulla oblongata for optimal lateral mass screw positioning when fusing the C1-ring.

**Materials and methods:**

Images of the cervical spine of 50 patients (64-line CT scanner) were evaluated and virtual screws were positioned in both lateral masses of the first vertebra using 3D-reconstructions of CT scans. The length of the screws, the insertion angles in two planes, the distance to the vertebral artery, and the spinal canal was investigated. Descriptive statistics was used and gender-dependent differences were calculated using student t-test. A diameter of 4 mm was chosen for the screws.

**Results:**

The mean screw length was 30.0 ± 2.3 mm on the right and 30.1 ± 2.1 mm on the left side. The arithmetic mean for the transverse angle was 16.4 ± 5.6° on the right and 15.6 ± 6.3° on the left, the sagittal angle averaged 8.3 ± 3.8° on the right, and 11.0 ± 4.9° on the left side. The mean distance between screw and spinal canal has been determined on the right with 2.4 ± 0.7 mm and 2.2 ± 0.6 mm on the left side. The distance from the C1 lateral mass screw to the vertebral artery was on average 7.1 ± 1.5 mm on the right side (significant correlation with gender, *p* value: 0.03) and 7.4 ± 1.4 mm on the left side.

**Conclusions:**

Screws should be positioned with a slightly converging angle of 16° and a slightly ascending angle of 10°. Due to the required high precision technique intraoperatively multiplanar 2 D or 3 D imaging is recommended to avoid harm to the vertebral artery or the spinal canal.

## Introduction

Burst fractures of the atlas, Jefferson fractures, only represent about 7% of all injuries of the cervical spine [1]. This type of fracture has an important meaning because incorrect treatment decisions in this sensitive region may have serious consequences to the patient; e.g., persisting pain, restriction in range of motion, or neurologic impairments [2, 3]. First and most important criterion in the choice of therapy is the question of stability. Stable fractures are usually treated conservatively while unstable burst fractures require surgery. The opinions on the proper surgical care of Jefferson fractures differ considerably, also reflected in surgical techniques [4, 5].

Posterior fusion with lateral mass screws is one of these techniques. This procedure must be planned very carefully. The screws have to be positioned exactly to avoid contact to the spinal canal and the risk of damaging the vertebral arteries with potential consecutive obstruction/aneurysm till stroke [6, 7]. A safe screw positioning is the best prevention avoiding complications. The purpose of this study was to determine safe zones regarding vertebral arteries and spinal canal for an optimal positioning of lateral mass screws.

## Materials and methods

We analyzed CT scans from 50 trauma patients that were examined with a whole-body CT. Twelve patients (24%) were female and 38 (76%) were male. The mean age was 44 years (male: 43.7 years; female 45 years). Images were recorded by a 64 slice Siemens SOMATOM Sensation^®^ CT system (Siemens Medical Solution USA Inc., 51 Valley Stream Parkway, Malvern, PA 19355, USA).

Anatomical measurements were performed using MIMICS^®^ image analysis software (Materialise, Leuven, Belgium). Data were analyzed using Microsoft Excel TM (Microsoft Excel 2003, Microsoft Headquarters, Redmond, Virginia, USA) and the Plug-In XL Statistics (Dr Rodney Carr, 14 MCGhie Road, Allanstford, VIC 3277, Australia). A p-value of 0.05 or less was considered statistically significant. To calculate the required space for the placement of two 4 mm screws, we used three dimensional models of 4 mm cortical screws (Hofer GmbH 6 Co KG, Fürstenfeld, Styria, Austria) and embedded them in our computer models.

**Fig. 1:**
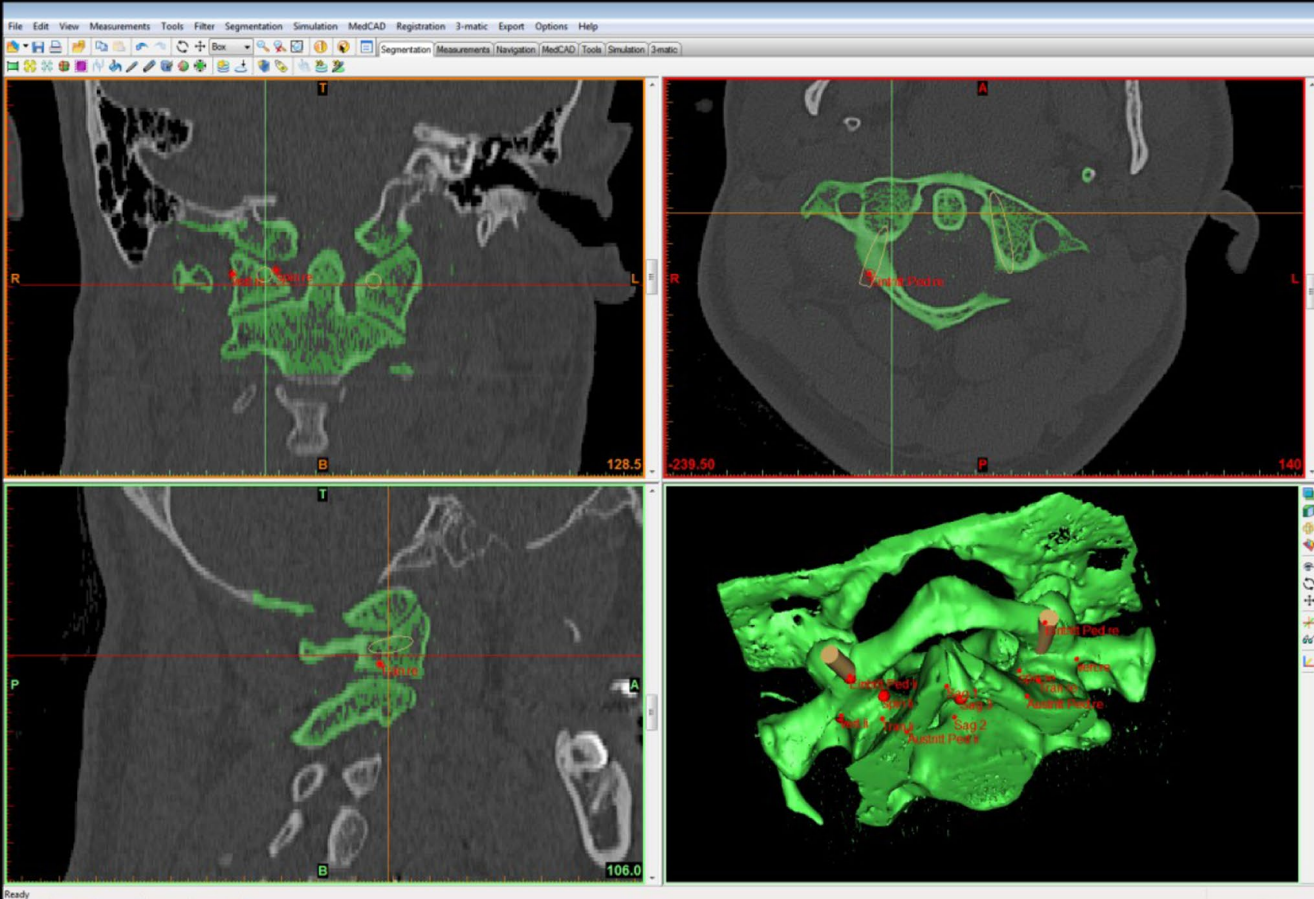
Screenshot with MIMICS^®^-Interface, multiplanar reconstruction

**Fig. 2:**
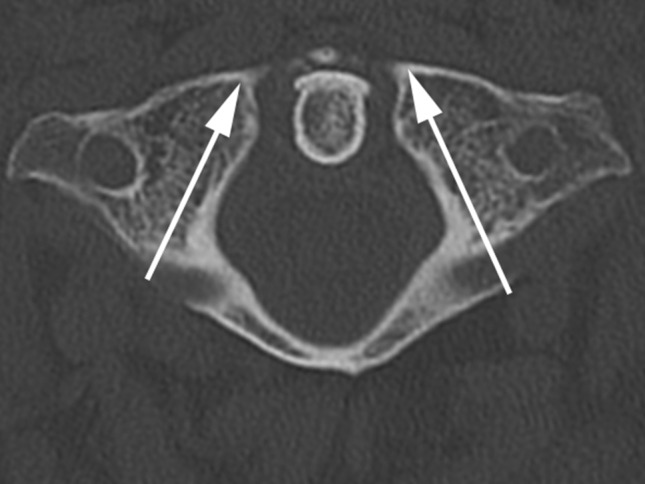
Technique for inserting the simulated screw (*arrows*) in transversal view

**Fig. 3:**
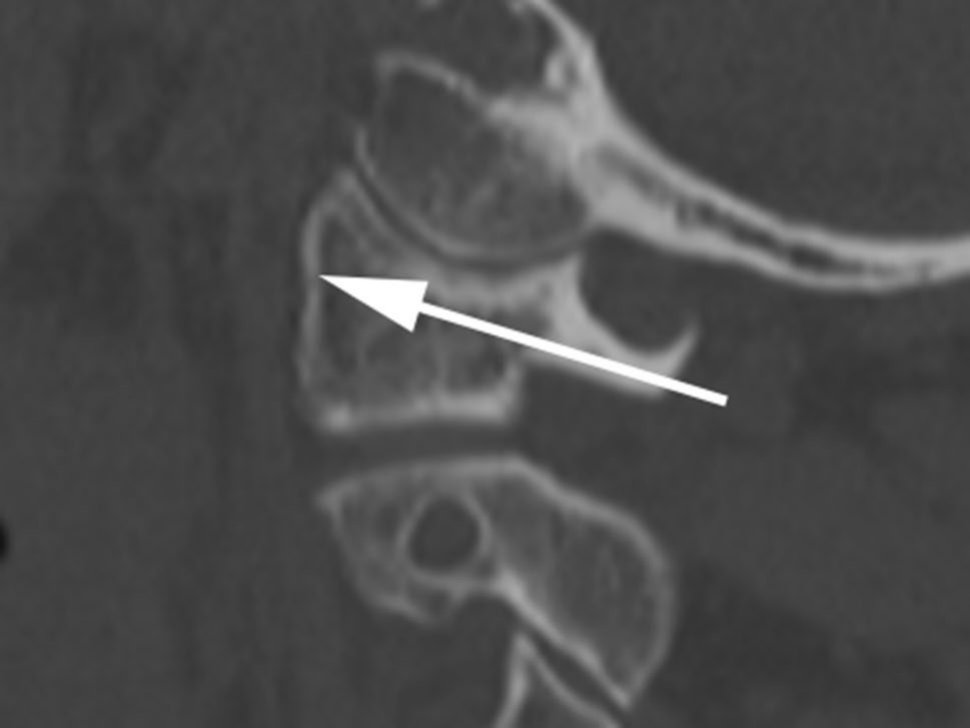
Technique for inserting the simulated screw (*arrows*) in sagittal view

First we defined the reference points for our measurements at the MIMICS^®^ interface, structured into a horizontal, frontal, and sagittal plane and also a 3D-view (Fig. 1). The different views allowed us to project lateral mass screws into the atlas in a correct position. The virtual screw was implanted in the lateral mass parallelly to the medial border of the atlas without penetration of the medial wall. We have chosen this slightly converging screw placement to protect the vertebral artery (Fig. 2). Furthermore, the lateral position of the screw head is less problematical when fusing the C1-ring instead of multi-segment stabilization (problems when screw heads are not in line). In sagittal plane, the screw was positioned parallelly to the arch avoiding penetration of the arterial groove. The screw axis had to be corrected when penetrating the C0-1 joint (Fig. 3). To determine the screw length, two points were set in the horizontal plane on both sides of the first cervical vertebra. The first point was set on the screw entry point and the second on the exit point. The distance between the two points corresponds to the screw thread length.

To measure the distance from the screws to spinal canal and vertebral arteries, one point was set in the spinal canal and the other in the foramen transversarium, both points were chosen narrowest to the screw. Then the distance between the two points and the screw was calculated.

**Fig. 4:**
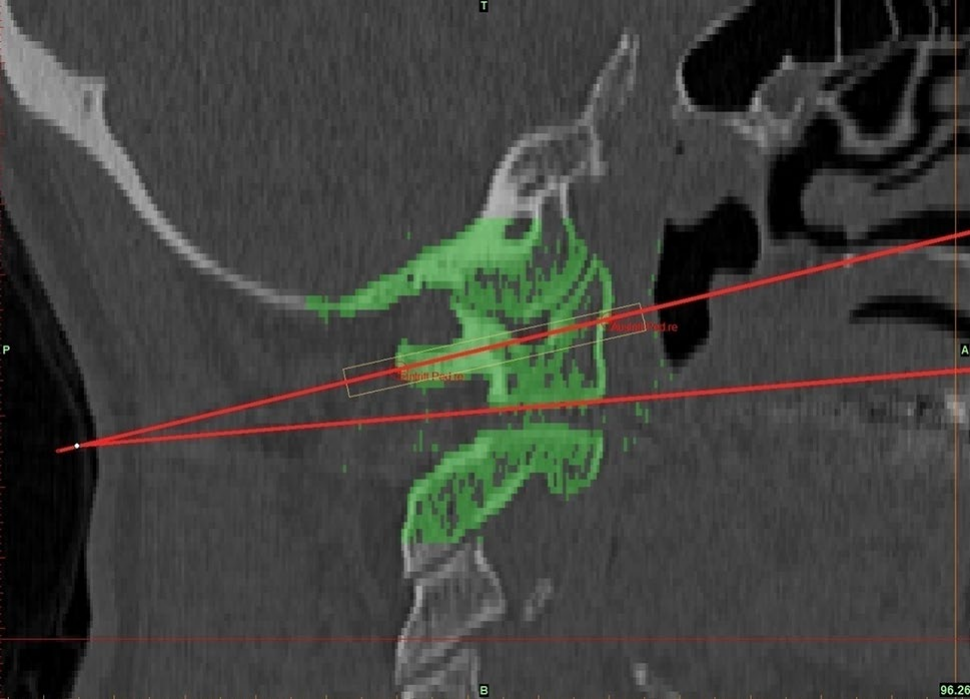
Measurement of sagittal insertion angle between simulated screw and base of C1

To determine the converging transversal angle, three planes were used. The first plane corresponds to the median plane, also known as body symmetry axis, and passes the anterior arc of the atlas, dens axis, and the posterior atlas arc. The other two planes were set at the screws vertically. The lines meet at the anterior atlas arc and include an angle, called transversal angle (Fig. 4). On sagittal image one line was drawn exactly at the base plate of the atlas. The other line corresponds to the screws entrance and exit point in sagittal view. The angle that is determined by these two lines is the screw entry angle, when the patient’s cervical spine is in the neutral position.

## Results

### Length of lateral mass screws

The average length, the distance between entry and exit point of the screw, is 30.0 mm (standard deviation ± 2.3 mm, relative standard deviation: 7.7%) at the right side and 30.1 mm (standard deviation ± 2.1 mm, relative standard deviation: 6.9%) at the left side. The median is 30.1 mm on the right and 30.5 mm on the left. The values range from 25.7 to 34.3 mm and 26.0–34.0 mm. The length of the screws showed no significant correlation, neither gender-specific (male *p* = 0.32; female: *p* = 0.37) nor side-specific (right side *p* = 0.18; left side *p* = 0.11); see Table 1.

**Table 1:** Length of lateral mass screw

Total	Mean (mm)	ơ (mm)	Rel. ơ (%)	Median (mm)	Min. (mm)	Max. (mm)
screw length, r	30.0	2.3	7.7	30.1	25.7	34.3
screw length, l	30.1	2.1	6.9	30.5	26.0	34.0

Male	Mean (mm)	ơ (mm)	Rel. ơ (%)	Median (mm)	Min. (mm)	Max. (mm)
screw length, r	30.3	2.2	7.3	30.3	25.7	34.3
screw length, l	30.1	2.1	6.8	30.6	26.0	34.0

Female	Mean (mm)	ơ (mm)	Rel. ơ [%]	Median (mm)	Min. (mm)	Max. (mm)
screw length, r	29.0	2.5	8.5	29.0	25.8	32.6
screw length, l	30.0	2.2	7.4	30.0	27.1	33.9

### Distance between screws, vertebral arteries, and spinal canal (safe zone)

The arithmetic mean for the smallest distance between screw and vertebral artery is 7.1 mm at the right side (standard deviation ± 1.5; relative standard deviation 21.2%). On the left side, this distance is 7.4 mm (standard deviation ± 1.4; relative standard deviation 18.5%). The median is 7.3 mm on the right and 7.6 mm on the left side of C1. There is a wide spread between the minimum and maximum value from 3.6 to 9.5 mm on the right side and 3.6–10.3 mm on the left side. Gender analysis of the results shows significant correlation between male gender and distance from lateral mass screws to vertebral artery at the atlas right side (*p* = 0.03) but no correlation to this on the left side (*p* = 0.10). Side-specific analysis reveals no significant correlation neither in male nor in female results (male *p* = 0.15; female *p* = 0.26).

The mean distance between the screw and the spinal canal is 2.4 mm (standard deviation ± 0.7 mm; relative standard deviation 30.5%) on the right side and 2.2 mm (standard deviation ± 0.6 mm; relative standard deviation 27.4%) at the left side. The median is 2.2 mm on the right and 2.1 mm on the left. Similar to the previous, the results are wide spread from 1.2 to 5.5 mm on the right side and 1.1–4.4 mm on the left side. There is neither a significant correlation to the distance between the lateral mass screw and the spinal canal and gender (male *p* = 0.09; female *p* = 0.29) nor a significant side-specific correlation (right *p* = 0.14; left *p* = 0.19); see Table 2.

**Table 2:** Distance between screws, vertebral arteries, and spinal canal (safe zone)

Total	Mean (mm)	ơ (mm)	Rel. ơ (%)	Median (mm)	Min. (mm)	Max. (mm)
Dist. to vert., r	7.1	1.5	21.2	7.3	3.6	9.5
Dist. to vert., l	7.4	1.4	18.5	7.6	3.6	10.3
Dist to spin., r	2.4	0.7	30.5	2.2	1.2	5.5
Dist to spin., r	2.2	0.6	27.4	2.1	1.1	4.4

Male	Mean (mm)	ơ (mm)	Rel. ơ (%)	Median (mm)	Min. (mm)	Max. (mm)
Dist. to vert., r	7.3	1.4	19.6	7.4	4.3	9.5
Dist. to vert., l	7.6	1.1	14.7	7.7	5.2	9.8
Dist to spin., r	2.4	0.8	33.3	2.3	1.2	5.5
Dist to spin., r	2.2	0.7	30.2	2.0	1.1	4.4

Female	Mean (mm)	ơ (mm)	Rel. ơ (%)	Median (mm)	Min. (mm)	Max. (mm)
Dist. to vert., r	6.4	1.6	24.8	6.7	3.6	8.6
Dist. to vert., l	6.8	1.9	27.9	6.9	3.6	10.3
Dist to spin., r	2.2	0.4	15.9	2.2	1.6	2.7
Dist to spin., r	2.2	0.4	16.8	2.2	1.5	2.9

### Transversal angle—horizontal insertion angle

The mean horizontal insertion angle is 16.4° on the C1 right side (standard deviation ± 5.6°; relative standard deviation 34.1%) and 15.6° (standard deviation ± 6.3°; relative standard deviation 40.1%) on the left side with respect to the body axis of symmetry. The median is 15.3° on the right and 14.9° on the left. Variations are shown from 7.1° to 33.5 on the right side and 5.5–34.4° on the left side. Gender- and side-specific analysis shows no specific correlation to transversal angle (male *p* = 0.30; female *p* = 0.43; right *p* = 0.92; left *p* = 0.65); see Table 3.

**Table 3:** Transversal angle–horizontal insertion angle

Total	Mean (°)	ơ (°)	Rel. ơ (%)	Median (°)	Min. (°)	Max. (°)
Angle trans., r	16.4	5.6	34.1	15.3	7.1	33.5
Angle trans., l	15.6	6.3	40.1	14.9	5.5	34.4

Male	Mean (°)	ơ (°)	Rel. ơ (%)	Median (°)	Min. (°)	Max. (°)
Angle trans., r	16.0	4.9	30.8	15.4	7.1	28.8
Angle trans., l	15.0	5.8	38.7	14.3	7.0	34.4

Female	Mean (°)	ơ (°)	Rel. ơ (%)	Median (°)	Min. (°)	Max. (°)
Angle trans., r	17.6	7.4	42.2	14.1	10.6	33.5
Angle trans., l	17.5	7.5	42.9	16.8	5.5	31.8

### Sagittal angle—sagittal insertion angle

The arithmetic mean for the sagittal insertion angle is 8.3° (standard deviation ± 3.8°; relative standard deviation 46.0%) on the right side and 11.0° (standard deviation ± 4.9°; relative standard deviation 44.5%) on the left in reference to the base plate of the atlas in a patient´s neutral position. On the atlas right side the median is 8.4°, on the left side 11.2°. Variations are measured between 0.8–14.9° on the right and 1.6–19.0° on the left side. Once more, gender- and side-specific analysis shows no specific correlation to transversal angle (male *p* = 0.06; female *p* = 0.43; right *p* = 0.87; left *p* = 0.997); see Table 4.

**Table 4:** Results, sagittal angle–sagittal insertion angle

Total	Mean (°)	ơ (°)	Rel. ơ (%)	Median (°)	Min. (°)	Max. (°)
Angle sagit., r	8.3	3.8	46.0	8.4	0.8	14.9
Angle sagit., l	11.0	4.9	44.5	11.2	1.6	19.0

Male	Mean (°)	ơ (°)	Rel. ơ (%)	Median (°)	Min. (°)	Max. (°)
Angle sagit., r	8.2	3.8	47.1	8.4	0.8	14.9
Angle sagit., l	10.3	4.5	43.4	10.6	1.6	19.0

Female	Mean (°)	ơ (°)	Rel. ơ (%)	Median (°)	Min. (°)	Max. (°)
Angle sagit., r	8.5	3.8	44.5	8.9	2.9	14.9
Angle sagit., l	13.3	5.6	42.4	16.0	1.9	18.3

## Discussion

Reviewing literature, there are several investigators reporting the surgical treatment of atlas fractures. However, the method using perpendicular planes to the screw thread is new. The prerequisite for this method is a 3-D-measurement software.

Concerning the screw’s thickness, diameters of 3–4 mm are described in literature. Screws with a diameter of 3 mm are commonly polyaxial screws.

The choice of the screw diameter depends on the nature of the fractured vertebra. Considering the position of the screw in the sagittal view (Fig. 3), it is noticeable that the screw ascends from the point of entry up to the anchorage in the ventral cortex. Male patients have an advantage concerning safe zones because they have a larger atlas. Using the above mentioned technique, our results illustrate, that lateral mass screws are closer to the spinal canal than to the foramen transversarium, contrary to the atlas pedicle screws. Comparing the distance between lateral mass screws and spinal canal or foramen transversarium of the male patient to those of the female, the measured distances in male patients were slightly higher.

Comparison with data from literature is difficult. Although a few studies describing the location of the lateral mass screw have been found, no equal technique was applied. Only the publication of Abeloos et al. contains detailed information on the nature and contribution of the screws [8]. Abeloos et al. reported about lateral mass screw with a diameter of 3 mm and a length of 24 mm compared with a mean length of 30 mm in our study [8]. We chose a diameter of 4 mm because 3.5 and 4 mm screws are commonly available for cervical spine instrumentation and a 4 mm monoaxial pedicle screw can be used from a thoracic spine system.

The transversal angle is described at a value of 20° [8], corresponding to our measured average value of approximately 16°. By contrast, Harms et al. reported about transversal angel in a range from 0° to 10° [9].

Measurements for the sagittal angle show a mean value of approximately 10° and a maximum of 20° in our study. However, comparing our data to those of Bühren, accordance is given [5]. Until today, there are no definitive guidelines to treat atlas fractures, surgically or conservatively. Several authors argue that even unstable Jefferson fractures heal with a sufficient immobilization [10–12]. In contrast, critics report about high rates of nonunion and persisting pain following conservative treatment [13, 14]. Atlas fractures appear unstable when the transverse atlantal ligament is ruptured [5, 10, 15, 16]. If there is proven ligamentous insufficiency, many surgeons recommend operative stabilization. There are several techniques to stabilize unstable atlas fractures [17–19]. Posterior C1 lateral masses fusion was described to be a very effective method. It has the advantage of a motion preserving technique to maintain physiological head rotation [8].

Our study shows the importance of pre-operative planning for posterior osteosynthesis of Jefferson fractures using lateral mass screws. Modern imaging and measurement software facilitate efficient and accurate representing of the local anatomy. Pre-operative dimensioning of the screws and planning of the screw entry points and angles are an excellent method to reduce screw mal-placement when intraoperative 3D-fluoroscopes are not available.
